# Measurement of luteinizing hormone surge in vaginal discharge: a potential biomarker that enables simple, non-invasive prediction of the periovulatory period

**DOI:** 10.1186/s12905-024-02916-4

**Published:** 2024-02-20

**Authors:** Moto Takeshita, Keita Saito, Yuya Suzuki, Wataru Yoshimasa, Risako Hayashi, Yoko Chiba

**Affiliations:** 1 Present Address: Cranebio Co., Ltd, 2-13-4 Sotokanda, Chiyoda-Ku, Tokyo, 101-0021 Japan; 2 Cellspect Co., Ltd, 2-4-23, Kitaiioka, Morioka City, Iwate 020-0857 Japan; 3 Present Address: Unicharm Co., Ltd, 3-5-19, Mita, Minato-ku, Tokyo, 108-8575 Japan; 4 Unicharm Co., Ltd, 1531-7, Wadahama Toyohama-cho, kanonji-shi, Kagawa 769-1602 Japan; 5 Department of Nursing, Kyoto College of Nursing, 1-21 Mibuhigashitakada-cho, Nakagyo-ku, Kyoto, 604-8845 Japan; 6 Present Address: Department of Midwifery, Graduate School of Human Nursing, The University of Shiga Prefecture, 2500 Hassaka-cho, Hikone City, Shiga 522-8533 Japan

**Keywords:** Fertile window, LH surge, Luteinizing hormone, Vaginal discharge

## Abstract

**Background:**

Predicting the periovulatory period is very important for conception. Current approaches to predicting the periovulatory period include monitoring of basal body temperature and urine luteinizing hormone (LH) concentration; however, these methods are time-consuming. Here, we examined the potential of using vaginal discharge (VD) as a non-invasive means of sample collection for determining the LH surge that indicates ovulation.

**Methods:**

Urine and VD samples were collected from 35 healthy women aged 20–39 years. VD samples were collected with panty liners to reduce the burden on participants. Daily first urine samples and used panty liners were collected from the 10th through 19th days of the menstrual cycle. Urine and VD LH (uLH and vLH) levels in the samples were measured by enzyme-linked immunosorbent assay. Measured vLH baseline and first surge values were analyzed using Student's t-test and ROC curves.

**Results:**

Samples for a total of 55 menstrual cycles were collected. We used uLH surge to establish the date of ovulation. uLH surges were observed in 49 cycles, 34 of which had corresponding VD samples that qualified for measurement. Five cycles were excluded due to a lack of vLH data. In the remaining 29 cycles, the vLH surge appeared within the fertile window 90% of the time, and the sensitivity and specificity of the test were 86% and 83%, respectively.

**Conclusions:**

VD has potential for use as a sample for predicting the periovulatory period by measuring LH content.

## Introduction

The fertile window is the period of time during the menstrual cycle when intercourse may result in fertilization. The window typically lasts a total of 6 days (the 5 days before ovulation plus the day of ovulation) and encompasses the time that the ovum is passing through the fallopian tube [1–3] Predicting ovulation is very important to increase the possibility of conception. The most commonly used biomarker for predicting the fertile window is urinary luteinizing hormone (uLH). LH is a hormone secreted by gonadotropic cells in the anterior pituitary gland that helps control the menstrual cycle. uLH level rises between 48 to 16 h prior to ovulation, which is known as the LH surge [4]. Although other methods of predicting ovulation are available, such as monitoring for an increase in basal body temperature (BBT) [5], the cervix mucus changes, the fern test [6], ultrasound imaging of ovarian follicles [7] and serum LH tests [4], the uLH test is simple to perform, has high predictive value, and is the only home test that can afford a positive result prior to ovulation [8]. However, the uLH test obliges users to collect a urine sample every day for 4–5 days or until a positive result is obtained, which can be a burden, even if a single test takes only 15 min [9]. Therefore, approaches to minimize the test workload are needed. Vaginal discharge (VD) is a mucus-based liquid that is constantly produced by the cells of the vagina and cervix. VD is naturally excreted outside of the body through the vagina, and it can be easily and non-invasively collected. We therefore hypothesized that should VD contain a detectable LH surge, then it would be possible to develop a non-invasive, hassle-free approach for predicting the fertile window. Similar concepts are already on the market for diapers that detect urination volume voided [10] and urine sugar content [11]. Here, we examined the use of VD as a sample for predicting the fertile window by measuring LH content. VD samples were collected on panty liners, and LH content (vLH) was determined by enzyme-linked immunosorbent assay. vLH was then compared with the uLH level in urine samples collected from the same women during the same menstrual cycle.

## Materials and Methods

### Sample collections and pretreatment

#### Participants

A total of 35 healthy, non-pregnant, Japanese women aged 20–39 years with a normal menstrual cycle (25–38 days) [12] were recruited to the study. All subjects were not obese (body mass index ≤ 25) and did not have any gynecological diseases, relevant past medical history, or medicine intake including oral contraceptives at recruitment. Recruitment was conducted through convenience sampling. In the case of candidates who had experienced childbirth, only those who were at least one year postpartum with a regular menstrual cycle were recruited. Thereafter, all the participants received a set of materials for urine and VD sample collection and envelopes for sample submission. Data and sample collection were conducted from March through June 2020.

#### Collection of personal information

Basic information about the participants was collected by means of a questionnaire form at the time of participation in the study. Age, weight, height, and childbirth history were collected from each subject. Information on the usual amount of VD and menstrual cycle such as the average number of days per cycle and days in bleeding were also collected.

#### Urine collection

Participants were required to collected their first urine every morning from the 10th through 19th day of their menstrual cycle. About 10 mL of urine was collected in a dropper bottle that was sealable with a screw cap), and then the primary receptacle was immediately placed in a leak-proof secondary package to avoid light exposure (a light-proof bag that could be closed completely) to avoid light exposure, and temporarily stored at 4 ºC in a home refrigerator.

#### Vaginal discharge collection

Participants were required to use the supplied surfactant-free panty liners (Unicharm Corporation, Tokyo, Japan). The VD was collected on the same day as the urine collection. At least one liner was used per day and also for 6–12 h to obtain sufficient VD. After use, the panty liner was immediately placed in a leak-proof primary receptacle (a Ziploc bag), with the bag lightly compressed to remove the inner air to prevent the liner from drying out, and then the primary receptacle was placed in a leak-proof secondary package (a light-proof Ziploc bag). If more than one liner was used, it was placed in a separate primary receptacle and placed in the secondary receptacle. The samples were temporarily stored at 4 ºC in a home refrigerator.

#### Submission of urine and VD samples by mail

Because the urine and VD samples were collected from healthy women, we considered there to be minimal likelihood that pathogens were present. Therefore, in accordance with World Health Organization regulations [13], the participants were asked to place the samples in three layers of packages for transportation to our laboratory. The first two layers were the receptacles described already in the Urine collection and Vaginal discharge collection sections above, and the third layer was an A4-size envelope lined with corrugated card. A postal service that allows packages to be delivered at a flat rate throughout Japan with a tracking service was used. In accordance with the World Health Organization regulations, the outside of the envelope included a clear description of the contents (“exempt human specimen”). The mailing address of the laboratory was also written, making it as easy as possible for the participants to send their samples. Upon mailing their samples, the participants were required to enter the tracking number of their package and the type and number of enclosed samples in a Google form to let the researchers know the delivery status. At the laboratory, the samples were stored in a freezer at − 20 °C.

#### Protein extraction from VD

A 2 × 2 cm square was cut from the center of each panty liner and placed in a 15-mL centrifuge tube. and placed in a 15-mL centrifuge tube. Then, 500 µL of 1% (w/v) protease inhibitor cocktail (CN:P8340; Sigma-Aldrich, St. Louis, USA) in 0.01 M phosphate buffered saline (pH 7.2–7.4; PBS; CN:162–18547; Fujifilm Wako Pure Chemical Corporation, Tokyo, Japan) was added to the tube. The VD sample was left to elute overnight at room temperature. The next day, the tube was centrifuged at 2430* g* for 20 min at room temperature and the protein extracts were transferred to 1.5-mL microtubes and stored at − 80 °C.

### Biotin-avidin enzyme-linked immunosorbent assay of LH

#### Preparation of anti-LH-beta antibody–coated plates

Anti-LH-beta antibody (CN:100,022; Oy Medix Biochemica Ab, Espoo, Finland) was diluted to a final concentration of 1 µg/mL with 50 mM aqueous sodium carbonate. Then, 100 µL of the LH-beta antibody solution was added to each well of a 96-well Nunc-Immuno Module plate (CN:444,865; Thermo Fisher, NY, USA) and the plate was incubated overnight at 4 °C. The coating solution was removed from the plate by washing three times with 300 µL of 0.05% Tween 20 in PBS. After washing, 300 µL of 1% Block ACE (CN:UK-B80; KAC, Kyoto, Japan) was added to each well and the plate was incubated overnight at 4 °C. The blocking solution was removed from the plate using a Multidrop™ Combi Reagent Dispenser (CN:5,840,300; Thermo Fisher) and the plate was left to dry overnight at room temperature.

#### Preparation of biotinylated antibodies

One hundred micrograms of anti-LH-alfa antibody (CN:100,066; Oy Medix Biochemica Ab) was diluted with 400 µL of 50 mM sodium hydrogen carbonate and filtered through an Amicon Ultra 0.5-mL Centrifugal filters (CN:UFC5003BK; Merck, Darmstadt, Germany) at 14,000 g for 10 min at 4 ℃. The residue on the filter was washed four times with 50 mM sodium carbonate. Then, 5 µL of 8 mg/mL Biotin-AC5-Osu (CN:72,040–63-2; Dojindo, Kumamoto, Japan) in *N*, *N*-dimethylformamide (CN:045–02916; Fujifilm Wako Pure Chemical Corporation) was added to the filter and the filter was incubated for 1 h at room temperature. The reaction was quenched with 40 µL of 1 M aqueous Tris–HCl (CN:35,406–75; Nacalai Tesque, Kyoto, Japan) and the reaction mixture was diluted with 260 µL PBS and centrifuged at 15,000 g for 10 min at 4 ℃. The residue was washed four times with 260 µL PBS, collected in a 1.5-mL tube, and diluted to 500 µg/mL to afford a biotin-labeled antibody solution, which was stored at 4 ℃.

#### Enzyme-linked immunosorbent assay

Urine was diluted to a final concentration of 5–50% with a dilution buffer composed of 1% Block ACE, 0.0745% EDTA, and 0.09% Proclin 950. A standard curve was generated with LH antigen (CN:996–31-1; Oy Medix Biochemica Ab) in diluent (0.0381, 0.0763, 0.153, 0.305, 0.610, 1.22, 4.88). VD extract was used for measurements in undiluted form. One hundred microliters of urine or VD extract was added to each well of a 96-well anti-LH-beta antibody–coated plate. The plates were incubated for 1 h at room temperature and then washed three times with 300 µL of 0.05% Tween 20 in PBS. Next, biotinylated anti-LH-alpha antibody solutions were diluted with the dilution buffer (1:200, v/v) and 100 µL of the solution was added to each well. After incubation for 1 h at room temperature, the plate was washed three times with 300 µL of 0.05% Tween 20 in PBS. A 1000-fold dilution of Streptavidin-Peroxidase Polymer, Ultrasensitive antibodies (CN:S2438; Sigma-Aldrich) with dilution buffer and 100 µL of the solution was added to each well and the plate was incubated for 1 h at room temperature. After incubation, the plate was washed three times with 300 µL of 0.05% Tween 20 in PBS, 100 µL of 3,3ʼ,5,5ʼ-tetramethylbenzidine Liquid Substrate System for ELISA was added to each well (CN:S1601; Agilent Technologies, Santa Clara, USA), covered with aluminum foil and the plate was incubated for 30 min. The reaction was stopped by addition of 100 µL of 1 M aqueous H_2_SO_4_ and the absorbance at 450 nm of each well was determined with a microplate reader (Mithras^2^ LB 943; Berthold Technologies GmbH & Co. KG, Bad Wildbad, Germany).

### Measurement of total protein (bicinchoninic acid [BCA] assay)

5 µl of VD protein extract was analyzed directly with a commercially available BCA assay kit (Takara Bio, Shiga, Japan).

### Measurement of urinary creatinine

Creatinine levels in urinary samples were measured with an Automatic Analyzer 3500 (Hitachi High-Tech Corporation, Tokyo, Japan), and IatroLQ, a creatinine assay kit (LSI, Tokyo, Japan), was used according to the protocols.

### Standardization of urinary LH

uLH levels vary due to the concentration of urine. To remove the concentration factor, urinary creatinine (Cr) was used as an index of the concentration of urine. The uLH levels were divided by the creatine levels (uLH/Cr) used in the analysis.

### Standardization of LH in VD

Since vLH levels were obtained as measurements of extracts of VD protein, vLH levels varied due to the amount of VD used for extraction, which was difficult to accurately quantify. Therefore, the amount of total protein (Pro) was used as an index of dissolved VD in extracts. The vLH levels were divided by the total protein levels (vLH/Pro) used in the analysis.

### Data analysis

The limit of detection (LOD) of each assay was calculated by means of the 3σ method [14]. If a measurement was lower than the LOD, the LOD was used instead, in accordance with a previous report [14]. If both uLH and Cr or vLH and Pro were lower than the LOD, the sample was excluded as “collection failed”.

The diagnostic value of the vLH was evaluated by Student’s *t*-test and receiver operating characteristic (ROC) analysis. Box plots were used to compare the distributions of the data baseline levels and first surge levels. A *p*-value of < 0.01 was considered to indicate statistical significance. The optical cutoff value for vLH in the ROC model was computed using the probability of detecting the positive cases that maximizes the Youden index. Statistical analyses were performed in Excel 2019 (Microsoft, Redmond, WA, USA) using BellCurve for Excel v4.04 (SSRI, Tokyo, Japan).

#### Determination of ovulation day

**Fig. 1:**
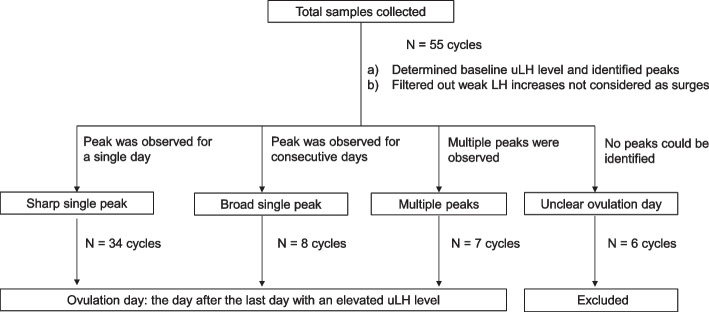
Process for determining the day of ovulation by measuring luteinizing hormone in urine (uLH)

Figure 1 shows an overview of the data processing. Ovulation day was defined as the day after the last day with an elevated uLH level, in accordance with a previous report [15], and was determined by graphing the change of uLH level over time. In the resulting uLH patterns, the baseline uLH level and uLH surges were identified. The baseline uLH level was determined as the average LH level for the 2 days before and 2 days after the peak maximum. Increases in uLH were counted as surges only when both of the following conditions were met: 1) uLH surge was greater than 30% of the maximum peak amplitude and 2) uLH surge was greater than two times the baseline LH level. Finally, the patterns were classified as showing a ‘sharp single peak’, ‘broad single peak’, or ‘multiple peak’. Patterns with no discernable peak were excluded.

#### uLH peak patterns

**Fig. 2:**
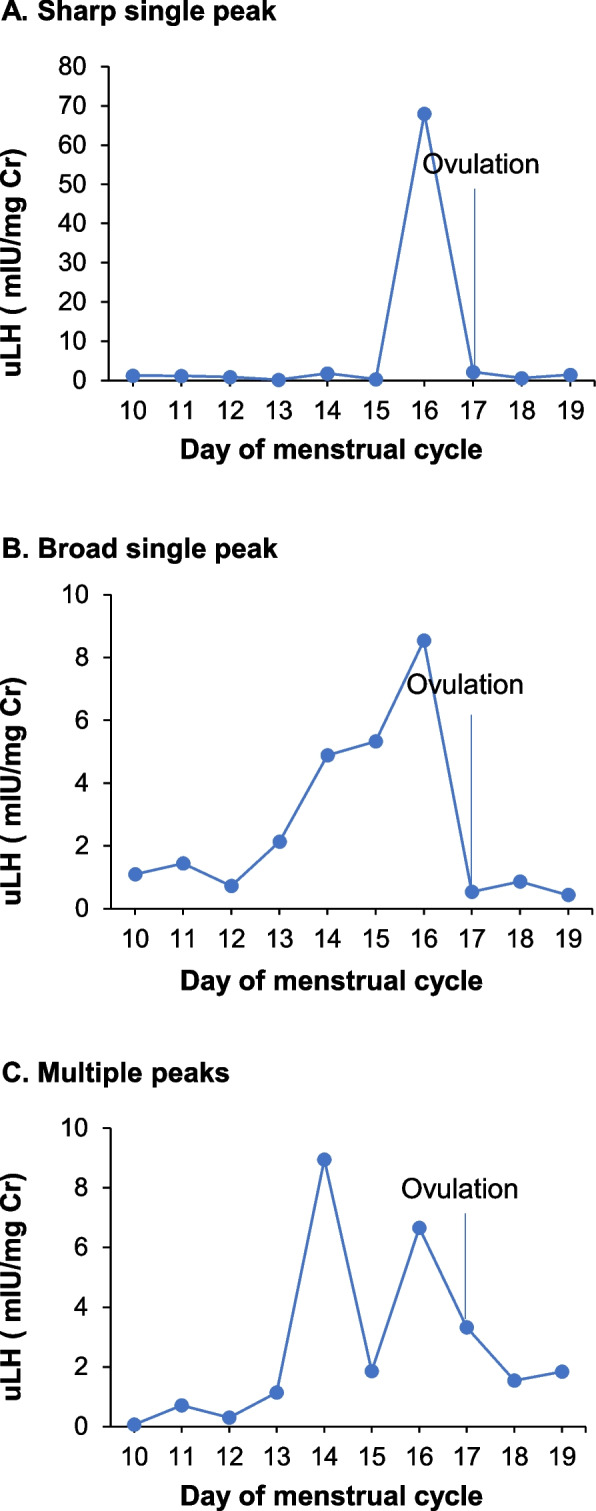
Representative urinary luteinizing hormone peak configurations. Days are counted from the first day of menstruation. The straight line pointed to by the word Ovulation indicates the ovulation day predicted from the uLH surge

The patterns with one or more peaks were further categorized into those showing a sharp single peak, a broad single peak, or multiple peaks (Fig. 2). In a pattern with a sharp single peak, uLH rapidly increased and peaked within one day and returned to baseline within the day following the peak. In this case, ovulation day was defined as the day following the peak. In a pattern with a broad single peak, uLH increased and remained elevated for several days before decreasing to baseline within the day following the peak. In a pattern with multiple peaks, several peaks were observed across several days. The broad single peak and multiple peak patterns are uncommon peak patterns [16–18] that occur because the LH surge does not always induce follicle rupture, and unruptured follicles do not provoke negative feedback for the production of LH [19]. For these two patterns, ovulation day was defined as the day after the final elevated day. The date of final elevated day was determined based on the definition of surge in the section above on determination of ovulation day.

## Results

### Participant characteristics

A total of 35 women participated in this study. Twenty of the women submitted urine and VD samples for two menstrual cycles, and the remaining 15 women submitted samples for one menstrual cycle; thus, samples from a total of 55 menstrual cycles were collected. The average age of the 35 participants was 32 years (SD, 5.6 years; range, 22–39 years), and the average body mass index was 20.6 (SD, 1.5; upper limit, 24.3). Seventeen participants were primipara, and eighteen were multipara. Average menstrual cycle length for the 35 participants was 30 days (SD, 2.4 days; range, 26–38 days).

### uLH peak patterns and the definition of ovulation day

For each of the 55 menstrual cycles, the daily uLH level during each cycle was plotted on a graph and the baseline and peaks were identified. The graphs for 49 of the cycles were classified as containing one or more peaks, with a peak defined as an increase then decrease of uLH to afford a complete peak shape. The remaining 6 cycles were excluded because no peak could be identified, either because the baseline could not be clarified or because insufficient sample was collected due to the participant not fully adhering to the protocol (Fig. 1).

### vLH peak patterns and appearance during fertile window

**Fig. 3:**
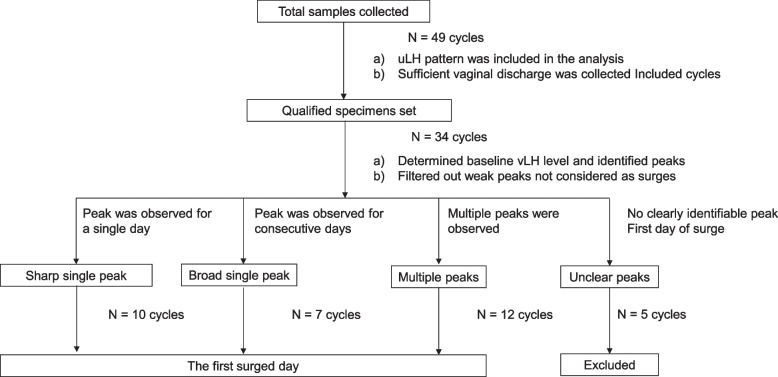
Process for determining the first surge day by measuring luteinizing hormone in vLH

Of the 49 cycles used for the uLH analysis, 34 had corresponding VD samples. Data processing similar to that applied to the uLH data was applied to the vLH data (Fig. 3). The vLH patterns for 10 cycles showed a sharp single peak, of which corresponding uLH peak patterns were 8 cycles showed a sharp single peak, 1 cycle showed a broad single peak and 1 cycle showed multiple peaks, 7 showed a broad single peak, of which corresponding uLH peak patterns were 2 cycles showed a sharp single peak, 4 cycles showed a broad single peak and 1 cycle showed multiple peaks and 12 showed multiple peaks, of which corresponding uLH peak patterns were 7 cycles showed a sharp single peak, 3 cycles showed a broad single peak and 2 cycle showed multiple peaks. Five cycles were excluded due to having no discernible peaks of which corresponding uLH peak patterns were 4 cycles showed a sharp single peak and 1 cycle showed multiple peaks.

In real-world uLH-based ovulation prediction, the first day of intercourse when a positive result is obtained is classified as the recommended day of intercourse. The same approach should be used for vLH-based ovulation prediction. The frequency of vLH-based recommended day of intercourse appeared within the fertile window is summarized in Table 1. And the fertile window was determined by using uLH surge. The vLH-based recommended day of intercourse appeared within the fertile window in 90% of the cycles examined, but the timing of its appearance within the window varied up to 5 days before the day of ovulation.

**Table 1:** Frequency of luteinizing hormone (vLH) surge in vaginal discharge within the fertile window

Timing of vLH surge	Number of cycles	Ratio
-5	7	24%
-4	4	14%
-3	2	7%
-2	8	28%
-1	3	10%
ovulation day	2	7%
others	3	10%
Total	29	100%

**Fig. 4:**
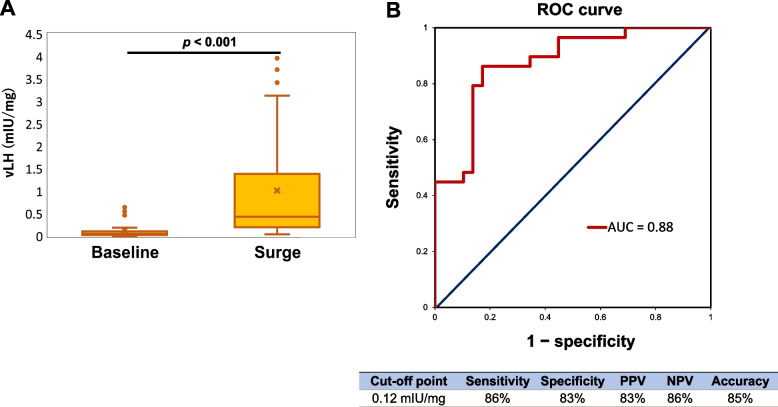
Statistical and receiver operating characteristic (ROC) analyses, and diagnostic parameters

To investigate whether vLH surge was sufficient surge or not, an ROC analysis and Student’s *t*-test were performed using the baseline and first day of surge vLH levels (Fig. 4A). The optimal cutoff value was 0.12 mIU/mg (according to the Youden index), and the sensitivity, specificity, positive predictive value, negative predictive value, and accuracy were 86%, 83%, 83%, 86%, and 85%, respectively. The area under the curve was 0.88. The result of the *t*-test was a *p*-value of < 0.01, which indicated statistical significance (Fig. 4B).

A) Box plot and *p*-value obtained from Student’s *t*-test. “Baseline” is the distribution of baseline values for each cycle. “Surge” is the distribution of vaginal discharge luteinizing hormone values (vLH) of the first surge that occurred in each cycle. B) ROC curve and area under the curve (AUC).

### Relationship between urinary (uLH) and vaginal discharge (vLH)

**Fig. 5:**
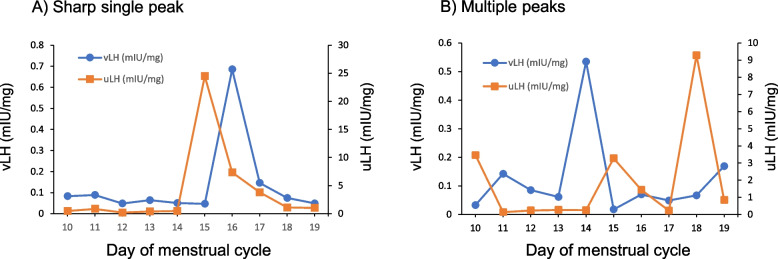
Relationship between urinary (uLH) and vaginal discharge (vLH) surge patterns. Each panel has a Shown a single cycle each. A) Strong surges occurred and returned to baseline within three days. The uLH preceded the vLH surge by one day. B) Weak peaks were observed three days prior to the strongest peaks. The vLH surge preceded the uLH surge by four days

Because both uLH and vLH originate from blood LH, uLH and vLH levels are theoretically related to one other. Although such a relationship was not observed for all of the menstrual cycles examined here, a strong relationship between uLH and vLH was observed for many of the participants (Fig. 5A). For the participants for whom no relationship was observed, we consider this to be due to insufficient sample collection leading to measurements being under the LOD. The correlation between uLH and vLH could not be described as a single relationship because in some participants one of the surges preceded the other by up to 4 days, whereas in others the two surges matched completely (Fig. 5B). Thus, compared with uLH, vLH seemed erratic, which affected diagnostic performance.

## Discussion

Here, we examined the possibility of using vLH as a biomarker to predict the fertile window. Since VD increases during the ovulatory period, we hypothesized that a sensor attached to a panty liner would provide the necessary amount of VD for the test, and we approached the measurement assuming actual use. VD was collected with panty liners to reduce the burden of specimen collection on participants; however, the amounts of VD were too little to recover by centrifugation. Extraction of protein from VD was able solve this problem, but quantifying the amount of dissolved VD became necessary. Assuming that the protein content in vaginal discharge does not change significantly, total protein content obtained by BCA assay was used, as described by Roberts et.al [20]. uLH is one of several methods for predicting ovulation day and the fertile window. Although some reports have mentioned difficulty in predicting ovulation with only uLH [21, 22], uLH is still the most accurate, non-invasive, and participant-friendly biomarker available. The performance of vLH was evaluated by two indexes: the timing of the surge and the strength of the surge. The percentage of menstrual cycles in which the first appearance of vLH surge fell within the fertile window was 90%, and the sensitivity and specificity of the test were 86% and 83%, respectively. These numbers were high enough and show that vLH is worth measuring, even if the performance is slightly inferior to that of uLH. However, further studies are needed to better understand the nature of vLH and to determine whether vLH is actually inferior to uLH. Ultrasonography was not performed in this study. In addition, we did not record basal body temperature or check the properties of cervical mucus, which have traditionally been used in conjunction with the measurement of urinary LH, so we were unable to determine the exact date of ovulation for each participant. Future research should also examine the secretion patterns of other gonadotropins in the discharge, FSH, and the ovarian steroids estrogen and progesterone in order to systematically define the characteristics of the vLH surge.

## Conclusion

Here, we investigated the possibility of using vLH as a biomarker to predict the fertile window. The first vLH surge appeared within the fertile window in 90% of menstrual cycles examined and the sensitivity and specificity of the vLH test was 86% and 83%, respectively. These results show the potential of using a built-in LH sensor in sanitary products as a hassle-free means of determining the fertile window. We expect that our findings will improve women’s quality of life.

## Data Availability

The datasets used and/or analyzed during the current study are available. from the corresponding author on reasonable request.

## Abbreviations

AUC: Area under the curve
BBT: Basal body temperature
FSH: Follicle stimulating hormone
LH: Luteinizing hormone
LOD: Limit of detection
ROC: Receiver operating characteristic
VD: Vaginal discharge
